# Promoting inclusivity in ecology, evolution, and behavioral biology education through course-based undergraduate research experiences

**DOI:** 10.1093/biosci/biae060

**Published:** 2024-07-30

**Authors:** Jake A Funkhouser, Megan Gregory, Crickette Sanz

**Affiliations:** Department of Anthropology at Washington University, St. Louis, Missouri, United States; Department of Evolutionary Anthropology at the University of Zurich, Zurich, Switzerland; College Writing Program and with the Center for Teaching and Learning at Washington University, St. Louis, Missouri, United States; Department of Anthropology at Washington University, St. Louis, Missouri, United States; Wildlife Conservation Society's Congo Program, Brazzaville, Republic of Congo

**Keywords:** diversity and inclusion, animal behavior, behavioral ecology, undergraduate education, field experiences

## Abstract

Access to independent research experiences is a persistent barrier that stifles the recruitment and retention of students from diverse backgrounds in ecology, evolution, and behavioral biology. The benefits of field experiences are not equitably available to historically excluded and minoritized students. In this article, we summarize evidence that indicates course-based undergraduate research experiences (CUREs) provide a solution to ensure equitable access to independent research experiences in the life sciences. We draw from our own experiences of teaching CUREs in ecology, evolution, and behavioral biology and provide the complete curriculum for our effective and largely materials-free CURE in behavioral ecology (CURE-BxEco). We advocate for greater access to and synthesize the benefits of CUREs to promote inclusivity in education. The proliferation of such innovative pedagogical practices benefits science because these classroom methods are critical in recruiting and retaining historically excluded and minoritized students, who offer diverse perspectives in research.

Diverse perspectives propel scientific innovation (Hofstra et al. 2020). However, access to independent research experiences in ecology, evolution, and behavioral biology has proven to be a persistent barrier to facilitating the inclusion of diverse perspectives in these fields.

The ability to participate in field courses and research *in situ* offers a range of personal, academic, and professional benefits (Beltran et al. 2020, Shinbrot et al. 2022, Hernandez et al. 2023). For most scientists in ecology, evolution, and behavioral biology, *the field* refers to the natural spaces where we learn to conduct science and from which data originate (Tydecks et al. 2016, Fleischner et al. 2017). Taking part in field research (i.e., field experiences) is often the first opportunity that students have to develop their skills in scientific literacy, independent research, and study design, which enables them to envision themselves as worthy practitioners of science (Beltran et al. 2020). In most of the life sciences, access to such field experiences is also critical for degree completion (Scott et al. 2012) and crucial to career advancement (McGuire et al. 2012). However, field courses and field experiences are not equitably available to students who have historically excluded, underrepresented, and minoritized identities (Cheyne 2019, Beltran et al. 2020, Tseng et al. 2020, Demery and Pipkin 2021), including students of color, LGBTQAI2S+, neurodiverse students, first-generation college students, migrant students, students with disabilities, and those with any combination of intersectional identities and backgrounds across these groups. We refer to the conglomerate identities of students rather than their whole selves to reflect the fact that underrepresented students often possess more than one identity that has been historically excluded and therefore minoritized in ecology, evolution, and behavioral biology (Veenstra 2011). The intersectional nature of their identities creates a unique combination of minoritized forces that students experience in context-specific conglomerates or universal sums, rather than in isolation (Emery et al. 2021).

Many minoritized students face obstacles to accessing independent research experiences in the field. Frequently cited barriers typically include a lack of time to engage in unpaid opportunities (Alwin et al. 2021), a lack of necessary funds and materials (Giles et al. 2020, Nunez et al. 2020), a lack of access to mentorship and support (Emery et al. 2019), and a lack of ability to travel to and safely navigate the contexts of *in situ* field experiences (Tseng et al. 2020). Unstructured outdoor settings pose a greater risk of unfamiliar environmental hazards that are difficult to traverse for students with disabilities (John and Khan 2018). Field experiences in outdoor rural localities are often challenging for BIPOC (Black, Indigenous, and people of color) and LGBTQAI2S+ students to safely negotiate, during both living and working in the field (Tseng et al. 2020). For example, minoritized students may face harassment, discrimination, and racism (Demery and Pipkin 2021), including local legislation that directly targets minoritized intersectional identities along the axes of sex, gender, and sexuality (Hughes and Kothari 2023). International field experiences can be equally dangerous to navigate for minoritized student communities. In fact, international travel is simply impossible for students of specific documentation, visa, or citizenship status. Even if possible, international field experiences are often accompanied by substantial financial obligations (Giles et al. 2020) and, in some nations, extreme legal precarity (e.g., anti-LGBTQAI2S+ legislation). Put simply, students with underrepresented, historically excluded, and minoritized identities often face a disproportionate set of barriers to accessing independent field research (Cheyne 2019, Demery and Pipkin 2021). The presence of these barriers suggests that there is a need to propagate other avenues for minoritized students to access research.

Immediate solutions are needed to provide equal opportunities for all students to access independent, inclusive research opportunities in ecology, evolution, and behavioral biology that are designed to ensure that no students are precluded from seeking access because of their underrepresented identities (Fleischner et al. 2017, Emery et al. 2019, Harris et al. 2020a, McGill et al. 2021). One strategy is to identify or create equitable, affordable, local, and safe field experiences that are designed with underrepresented, historically excluded, and minoritized students in mind (Demery and Pipkin 2021, Rudzki et al. 2022). Although it is a necessary step forward, even field experiences that strive to be inclusive are often entangled with complicated, exclusionary ideologies, and local beliefs that preclude minoritized students from striving to reach the field at all because they feel that they will be unwelcomed in these spaces (John and Khan 2018, Emery et al. 2021).

There are three key challenges to offering inclusive access to field experiences for all students: piquing minoritized students’ interest in disciplines that place an intellectual emphasis on field experiences (Mourad and Middendorf 2022), ensuring that interested minoritized students who are unable to access the field are able to see themselves as worthy participants in historically field-based disciplines (O'Brien et al. 2020), and providing equitable opportunities for minoritized students to access independent research that is necessary for academic and career advancement (Schell et al. 2020, Totonchi et al. 2021). Given that coursework is a universal component of university education (Handelsman et al. 2022), we suggest that it provides an often overlooked potential solution to the obstacles that hinder inclusive field experiences.

Fully leveraging the potential of inclusive pedagogy in university courses can aid in overcoming systematic disparities in the academic achievement of historically excluded and minoritized students compared with their majority counterparts. Active learning is one example of inclusive pedagogy that has been shown to be an effective strategy for narrowing the achievement gaps of minoritized students (Preszler 2009, Haak et al. 2011, Theobald et al. 2020) and increasing retention in science (Harris et al. 2020b). Many studies on active learning have illuminated its effectiveness compared with what happens in more passive environments, such as classrooms dominated by traditional lecturing (Handelsman et al. 2022). Course-based undergraduate research experiences (CUREs) are a type of active learning pedagogy that offers a suite of positive benefits to students, including increased scientific literacy (Valliere 2022), science self-efficacy (McBride et al. 2020), a sense of belonging in science (Connors et al. 2021), and access to inclusive independent research opportunities (Smith et al. 2023). In the present article, we illustrate the potential of CUREs to promote inclusive education in ecology, evolution, and behavioral biology. We use our experiences teaching hybrid forms of a materials-free CURE to guide our analysis. In addition, we provide our complete curriculum to our CURE in behavioral ecology (CURE-BxEco) to promote the proliferation of such approaches and evidence-based examinations of whether such CUREs are effective in achieving inclusive change.

## CUREs can provide inclusive access to authentic research

**Figure 1.**
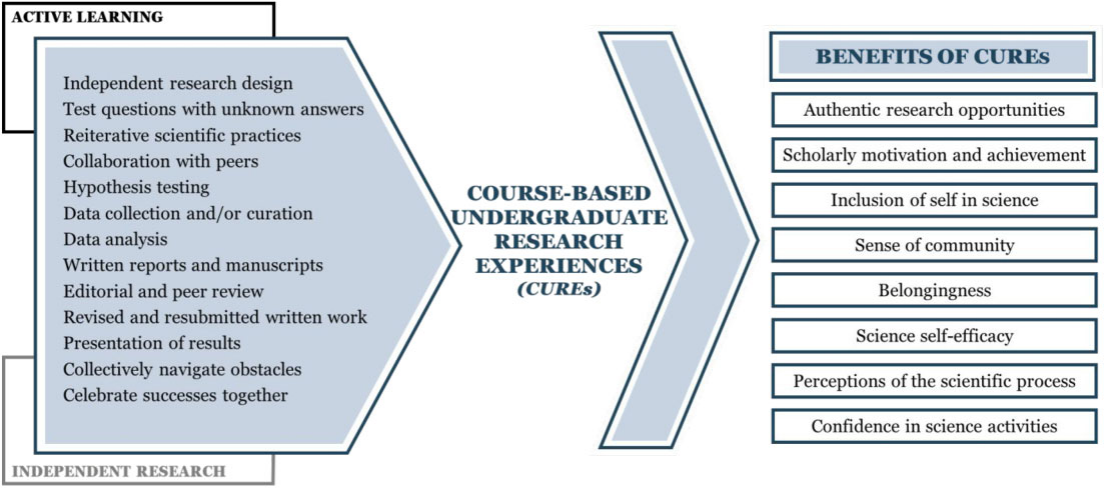
The components and benefits of course-based undergraduate research experiences (CUREs).

In CUREs, students engage in reiterative scientific practices to address research questions, collaborate with peers, and discover the relevance of their work outside of the classroom (figure 1; Buchanan and Fisher 2022). CUREs provide students with the agency not only to engage in scientific activities but to do so authentically by defining their own research focus through independently designing studies that investigate research questions with currently unknown answers (Brownell et al. 2012, Ballen et al. 2017a). For example, within a single semester, students can engage with primary literature to develop an original research question, identify appropriate methods, collect or curate data, test hypotheses, and learn to effectively communicate results through the academic process of revising and resubmitting written work after peer review (Fey et al. 2020).

In addition, CUREs have the potential to provide research experiences to a greater number of students than would be possible in conventional mentoring opportunities (Smith et al. 2023) as part of the same successful mentorship framework (Emery et al. 2019). In multiple biological sciences programs, the total number of students who had access to independent research experiences more than doubled with access to CUREs compared with one-on-one research mentorship models (Stanford et al. 2017, Jones and Lerner 2019, Smith et al. 2023). Unlike other experiential pedagogical frameworks, which can be cost prohibitive, CUREs that focus on behavioral observations of local animals or other scientific endeavors that do not require many physical materials can exist outside of external funding streams, making these learning experiences accessible to any institution, degree program, and instructor (Sorensen et al. 2018, Jones and Lerner 2019, Gastreich^ 2021^, Sorensen et al. 2021, Tripepi and Landberg 2021). Accounting for students’ varying access to financial and physical resources has the potential to advance efforts to provide equitable and inclusive independent research opportunities across the evolutionary, ecological, and behavioral sciences.

## CUREs can disproportionately benefit underrepresented students

CUREs offer many enhanced learning and achievement opportunities to students via the composite benefits of several active learning concepts, including experiential learning (Kong 2021, Stock and Kolb 2021), project-based learning (Eberlein et al. 2008), case-based learning (Holland and Pawlikowska 2019), collaborative learning (Wilson and Varma-Nelson 2019), and learning by doing (Metzger and Langley 2020). CUREs have also been demonstrated to improve students’ critical thinking skills (Brownell and Kloser 2015) and to effectively teach students to engage in the scientific process (Gin et al. 2018).

In addition to nurturing the scientific literacy of those students who plan to pursue careers in science (Millstone and van Zwanenberg 2000, Roberts et al. 2013, Sharon and Baram-Tsabari 2020, Wintterlin et al. 2022), all students can benefit from enhancing their skills in applying scientific concepts to evaluate claims about the world around them (Shaffer et al. 2019). Although scientific literacy commonly improves with engagement in science, historically excluded and minoritized students typically have less experience with science before reaching university science courses. Therefore, curricula that nurture students’ scientific literacy skills can help level the playing field by disproportionately benefiting students from minoritized backgrounds (Theobald et al. 2020). Furthermore, research experiences in CUREs regularly culminate in student-authored research reports, which have been demonstrated to build critical scientific literacy skills, to benefit students’ self-esteem, and to aid in clarifying their personal and professional goals (Theobald et al. 2020, Turner et al. 2021). If not for CUREs, underrepresented and minoritized students may not have opportunities to otherwise engage in research activities and produce manuscripts that are critical for their professional advancement (Shortlidge et al. 2017).

Developing the scientific literacy skills of historically excluded and minoritized students is especially critical given their sense of belonging in the scientific enterprise is often stifled by the disproportionate burden they face in dismantling historically racist and exclusionary forces (O'Brien et al. 2020). Therefore, providing minoritized students with the skills to deconstruct racial oppression and White supremacy (Schell et al. 2020), address misconceptions about natural selection and evolution (Mead et al. 2018), and dispel dichotomous caricatures of human variation in gender, sex, and sexuality (Zemenick et al. 2022) is essential to promoting an inclusive and diverse future for the biological sciences. CUREs are an exceptionally promising avenue to improve students’ scientific literacy skills while disproportionally benefiting the learning and inclusion of historically excluded and minoritized students.

## CUREs can provide students with a sense of belonging in science

Not only do CUREs improve the scientific skills of historically excluded and minoritized students so they *can* engage in science, but CUREs can also change students’ abilities to *believe* that they are worthy practitioners of science. Science self-efficacy is an individual's belief in their capabilities to engage in scientific inquiry (Bandura 1977, McBride et al. 2020). Increasing science self-efficacy for students has been suggested as a fundamental step in recruiting and retaining scholars from underrepresented and historically marginalized backgrounds in STEM (Robnett et al. 2015, Ballen et al. 2017b). Engaging students in the scientific process in general education courses significantly increases their self-efficacy and connection of their selves to science (McBride et al. 2020). Importantly, the greatest increases in self-efficacy were for minoritized and international students.

By increasing students’ self-efficacy, CURE pedagogy also increases students’ sense of belonging in science (Turner et al. 2021). Belonging includes perceptions of connectedness, social inclusion, and the construction of meaningful social relationships with peers and mentors in scientific contexts (Fink et al. 2018). Providing students with the opportunity to construct a sense of belonging in science is critical to retaining diverse perspectives in any scientific discipline (O'Brien et al. 2020).

A sense of belonging within specific courses, as opposed to campus-wide or discipline-specific belonging, has been suggested as the most influential source of support for the behavioral and emotional engagement of underrepresented and historically excluded STEM students (Wilson et al. 2015, O'Brien et al. 2020). CUREs therefore offer a critical avenue for fostering a sense of belonging by inviting students to participate and contribute to a research network (Connors et al. 2021, Turner et al. 2021). The benefits of belonging offered by CUREs are greatest for students from historically underrepresented backgrounds in science (Chemers et al. 2011, Fink et al. 2018, Syed et al. 2019). The communities constructed in CUREs can be effective in generating a greater sense of self-efficacy and scientific belonging that have cascading benefits to student personal growth, career development, and disciplinary contributions (O'Brien et al. 2020).

## CUREs in ecology, evolution, and behavioral biology

The benefits of CUREs in ecology, evolution, and behavioral biology have been repeatedly demonstrated (see table 1) and can be highlighted through a few case studies. For example, in one CURE, students independently studied canid behavioral ecology and this experience provided them with a sense of place during the uncertain times of a global pandemic (Valliere 2022). In a similar course, students developed advanced scientific skills while engaging in, what they recounted as, meaningful real-world research and reported being more likely to enroll in graduate programs and seek employment related to science as a result of participating in the CURE (Sorensen et al. 2018). Although there are many examples of CURE curricula in the life sciences (e.g., CureNet, serc.carleton.edu/curenet), there are few that take our approach in combining research experience with materials-free data collection and writing-intensive training in scientific literacy.

**Table 1.** Benefits of CUREs in ecology, evolution, and behavioral biology.

CURE	Setting				Learning outcomes	Reference
Species distribution and biodiversity of urban birds	M			F	Scientific literacy, advocacy for urban conservation, participation in eBird	Gastreich 2021
GIS investigations with open-source ecology and environmental data		C			Scientific competency, experience working in collaborative remote teams	Fey et al. 2020
Species distribution and mammalian community dynamics using camera trap methods				F	A sense of place and belonging, the ability to advocate for and communicate science	Sorensen et al. 2021, Tripepi and Landberg 2021, Valliere 2022
Coastal dune plant community restoration				F	Scientific literacy, systems thinking, greater sense of science identity	Stanfield et al. 2020
Behavioral ecology of goats		C	O		Community engagement, international collaboration, critical thinking	Kay et al. 2023
Behavioral ecology of squirrels (Squirrel-net)			O	F	A sense of community, career-readiness skills	Connors et al. 2021
Behavioral ecology of animals (CURE-BxEco)	M	C	O	F	Science self-efficacy, writing intensive, a sense of belonging in science	The present article

*Note:* The setting of each CURE refers to modules conducted in a single class session (M), independent projects conducted from the classroom or exclusively during scheduled class time (C), independent projects conducted using online resources (O), and urban or local field experiences that were conducted outside of the classroom and outside of specifically scheduled class time (F).

## A new CURE in behavioral ecology (CURE-BxEco)

Our institution has offered a materials-free, accessible, and effective CURE since as early as 1965. We call this curriculum “Course-Based Undergraduate Research Experience in Behavioral Ecology” (CURE-BxEco). The course is an advanced-level, writing-intensive seminar that provides students with a semester-long, course-based research experience, which also fulfills a general education requirement in the College of Arts and Sciences.

**Figure 2.**
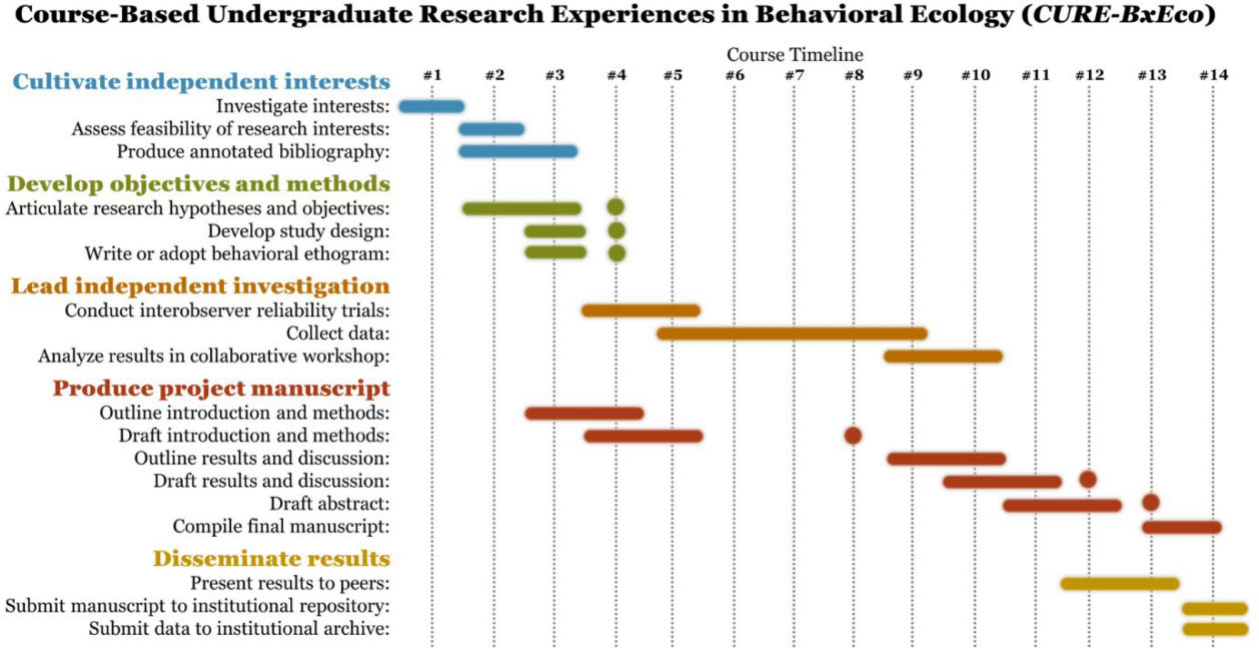
Overview of CURE-BxEco curriculum components and week-by-week timeline. Bars indicate the time students spend independently working on these components of their projects. All students receive feedback after their initial submission. Dots indicate targeted submission dates for revised or updated assignments.

The course curriculum enables instructors to both guide and mentor students as they conceptualize, design, and conduct research projects that include 4 weeks (more than 40 hours) of independent data collection on animal behavior (figure 2). During class sessions, lecture materials and activities are designed to equip students with a background in behavioral and ecological theory, as well as state-of-the-art methods for observational behavioral data collection that can be employed in their projects, such as using customizable apps for data collection (Ross et al. 2016). Students also engage in peer-review workshops to review their writing and work collaboratively to establish interobserver reliability in the methods of each of their projects. Each week, students receive individualized feedback and mentorship on their research designs, drafted manuscript sections, data collection, and research findings. By the end of the semester, all students produce a complete manuscript written in academic journal style and share their findings as an academic conference presentation.

**Figure 3.**
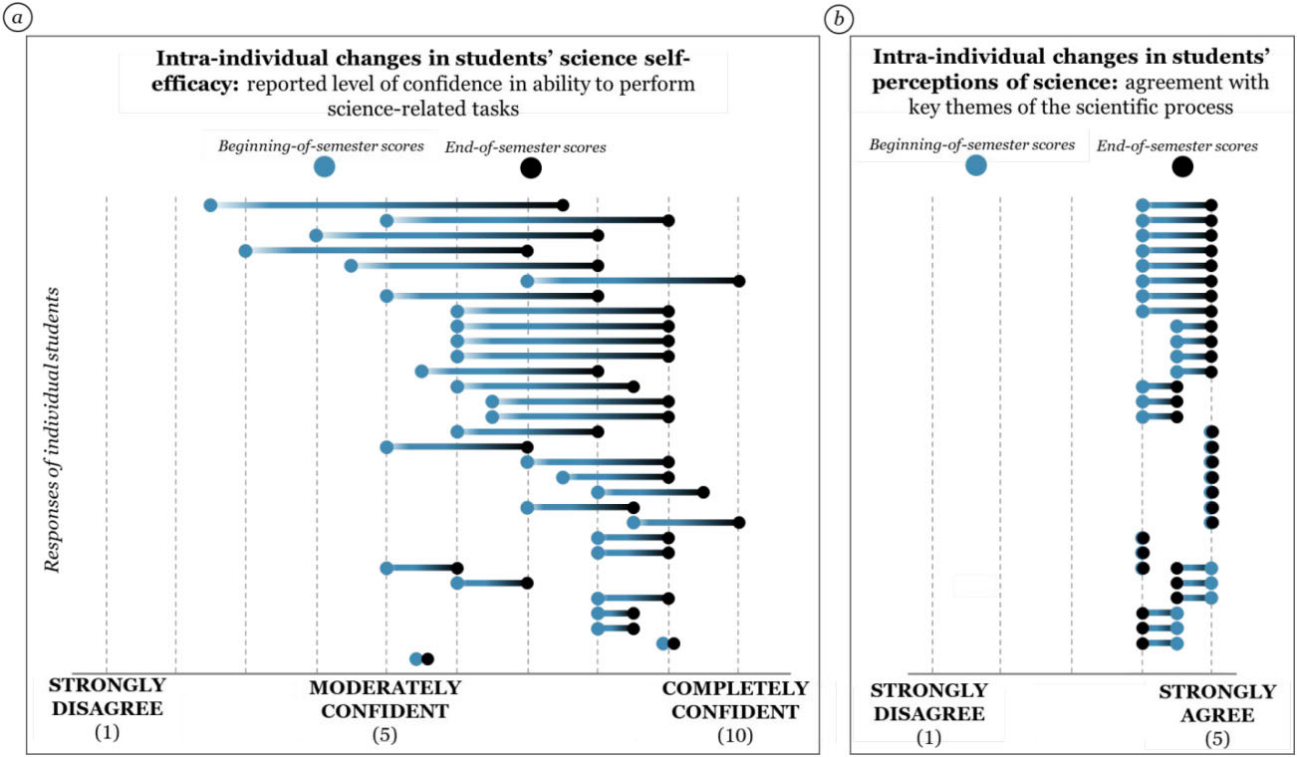
Change in median composite responses of individual students to the (a) Science Self-Efficacy Scale and (b) Perceptions of Science Scale. Each pair of points represents the response of a single student. Science self-efficacy was measured using McBride and colleagues (2020) items where students were asked to rate their confidence (on a scale of 1–10) in performing several science-related tasks. We measured students’ perceptions of science by asking them to rate their agreement (on a scale of 1–5) with several statements related to the inclusive and realistic nature of the scientific process. Both measurement scales are reported in supplemental tables S4–S5.

CURE-BxEco and similar course-based research opportunities carry the most impact for underrepresented and minoritized identities. By fostering students’ science self-efficacy and sense of belonging in science classrooms, CUREs can contribute to the persistence of underrepresented and minoritized identities in science. Our CURE-BxEco curriculum has been documented to be effective in these areas. Participation in CURE-BxEco significantly increased students’ self-reported science self-efficacy, changed their beliefs about science, and improved students’ sense of belonging in science (figure 3; also see the supplemental material).

**Figure 4.**
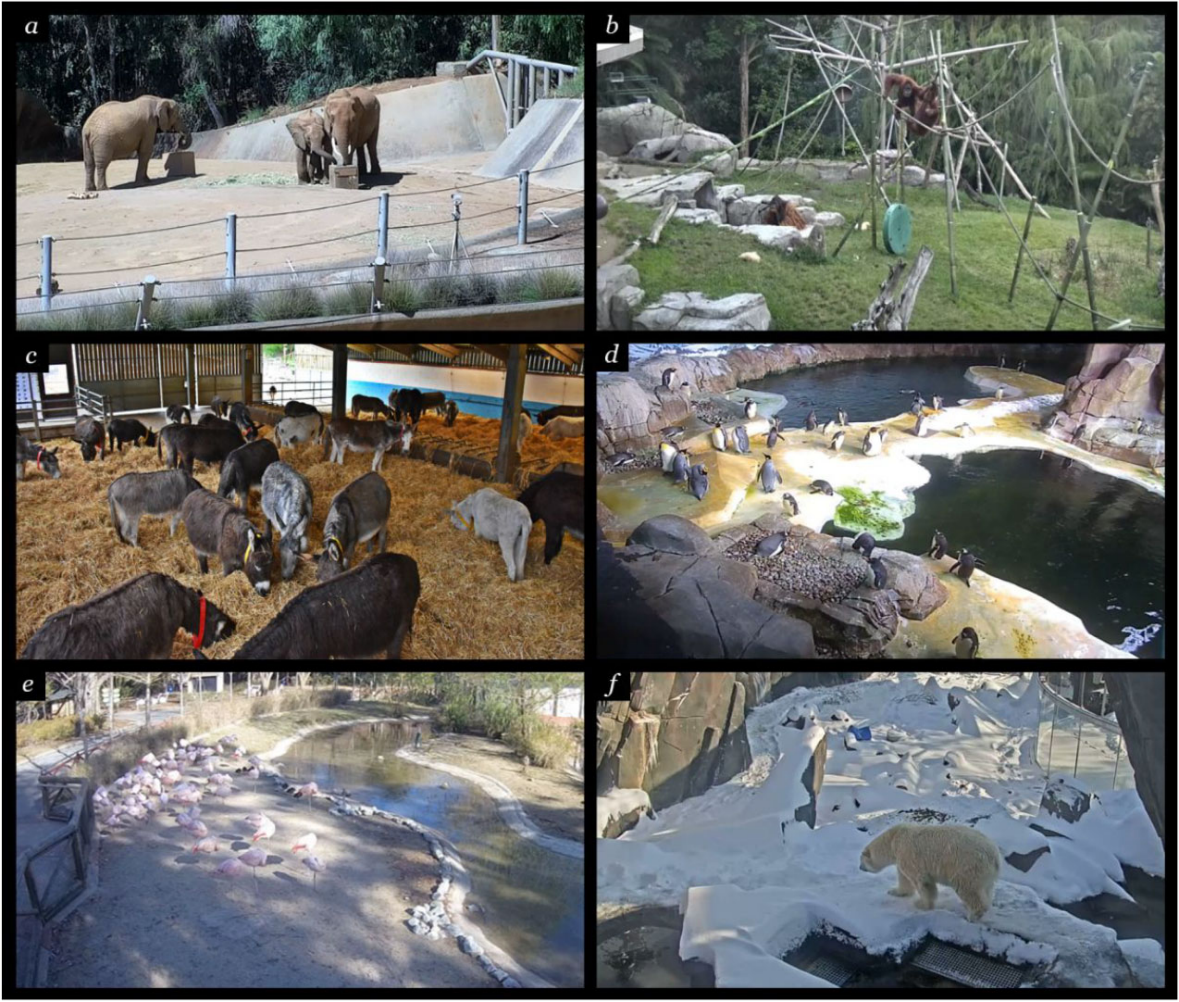
Examples of remote data collection via live animal webcams that students of CURE-BxEco used in their independent research projects: (a) elephants at San Diego Zoo Safari Park (https://sdzsafaripark.org/cams/elephant-cam), (b) orangutans and siamangs at San Diego Zoo (https://zoo.sandiegozoo.org/cams/ape-cam), (c) domestic donkeys at The Donkey Sanctuary (https://thedonkeysanctuary.org.uk/webcams), (d) penguins at Kansas City Zoo (https://kansascityzoo.org/animal-cam/penguin-camera), (e) flamingos at Maryland Zoo (https://marylandzoo.org/animals/live-cams-feeds), (f) polar bears at Saint Louis Zoo (https://stlzoo.org/animals/live-animal-webcams). A database of possible live animal webcams can be found at https://congo-apes.org/CUREBxEco.

Although students had historically conducted data collection via direct observation at the Saint Louis Zoo, this was not possible during the COVID-19 pandemic, and so we adapted the course curriculum to provide students with additional opportunities to conduct their research using live-streamed video webcams and video databases that are freely available. Examples of animal webcam views successfully used by our students are included in figure 4 and supplemental table S1. When the courses returned to in-person instruction, we continued to integrate opportunities for live observations, remote observations, and data collection from video archives into the course curriculum so as to maximize our flexibility in accommodating the diverse needs of students and to enhance the breadth of research opportunities provided by CURE-BxEco. In doing so, the students were afforded new possibilities to increase their sample sizes, conduct comparative investigations across species or study sites, and screen footage for behaviors that are rare or difficult to score during real-time observations.

By incorporating freely available animal webcams, the components of CURE-BxEco are more widely portable and can be implemented across many educational settings, therefore extending research experiences to a greater number of historically excluded and minoritized students. Furthermore, by integrating an open science initiative and launching an associated repository for data sets related to this curriculum, we hope to both provide open scholarship training for our students as well as catalyze expansive and comparative research by other scholars. A copy of our entire curriculum and animal webcam database is provided at https://congo-apes.org/CUREBxEco.

CURE-BxEco has been successful in providing students with opportunities to engage in authentic research experiences with unique combinations of questions and study populations. On several occasions, projects stemming from this course have resulted in published, peer-reviewed manuscripts. For example, data on black-tailed prairie dog behavior, response to visitors, and enclosure use have informed the care and welfare management of this species (Eltorai and Sussman 2010, 2011). In another project, data on the social interactions of bachelor gorillas were collected by multiple students over several semesters. These data and their results offered relevant insights into the care and management of western lowland gorillas (Gartland et al. 2018). The type of collective and longitudinal data collection activities associated with CURE-BxEco offers untapped possibilities to address the need for data in zoo science, documenting taxonomic behavioral diversity, advancing conservation biology, and improving the management of captive animal well-being (Cronin et al. 2021, Garcia-Pelegrin et al. 2022).

In addition, the pressing need for research in the zoological, conservation, and well-being sciences assures students that their research is meaningful and relevant in many ways outside of the classroom. Therefore, CURE-BxEco can amass much-needed information and data archives that could span a considerable variety of taxa, number of individuals, and temporal depth. We look forward to seeing a greater diversity of live animal webcams, both in terms of the number of species and the types of institutions providing live webcam views in the coming years. We encourage institutions to invest in publicly accessible webcams and for studies to be conducted to understand the impact of such resources. Furthermore, we hope those with webcams will see the value of providing students or the public with on-screen date and time information and web access to archives of previous footage along with links to group size, demographics, individual life histories, and identifying characteristics of individuals. Reciprocally, we encourage students and educators who use live animal webcams to share their use, impact, study details, and the results of their course-based investigations with curators and caregivers at the host institution.

CURE-BxEco is novel in ecology, evolution, and behavioral biology because it combines previously disparate pedagogical components: an exhaustive database of animal webcams as a complement to in-person data collection to provide greater access to semester-long independent research experiences and is centered around writing-intensive components to strengthen career-readiness skills and professional advancement. By facilitating personalized opportunities for students to engage in research and contribute novel findings to academia, CUREs also have the potential to enhance the equity and inclusion of students with underrepresented identities across disciplines. This curriculum is also effective in promoting students' science self-efficacy and sense of belonging, while offering opportunities for academic and professional advancement (supplemental tables S2–S3). Both science self-efficacy and greater opportunities for advancement are mechanisms that contribute to the persistence of underrepresented and minoritized students in the sciences (Ballen et al. 2017a, McBride et al. 2020).

## The promising future of CUREs

CUREs can leverage the documented benefits of active learning while providing students with authentic, original research opportunities to be scientists. Engaging in a CURE increases students’ confidence to engage in scientific activities (McBride et al. 2020, Stanfield et al. 2022). Thereby, not only is CURE pedagogy effective in providing students with the skills they need for a productive career in science (Shaffer et al. 2019), but CUREs also have the potential to change the ways students think about science and themselves as scientists (Ballen et al. 2017b, Connors et al. 2021), while providing greater access to independent research opportunities (Jones and Lerner 2019, Smith et al. 2023). However, CUREs are just one among many mechanisms that are necessary to truly propel an inclusive and diverse future of ecology, evolution, and behavioral biology. For example, novel recruitment and retention strategies are necessary to overcome the systematic barriers that historically excluded and minoritized students face in accessing college- and university-level education in ecology, evolution, and behavioral biology.

In addition to intervention programs aimed at ensuring that these students gain university admissions and financial assistance, CUREs taught by university faculty to secondary students in high schools with limited resources have the potential to enhance the ability of historically excluded and minoritized students to envision themselves as scientists. Providing precollege students with access to the benefits of CUREs has broad potential to break down systematic barriers and pique the scientific interests of historically excluded and minoritized students in ecology, evolution, and behavioral biology (O'Brien et al. 2020, Mourad and Middendorf 2022). We designed the CURE-BxEco curriculum and course resources to be not only accessible but easily adaptable to different education levels and generalizable to any field that uses the scientific process. Educators from high schools to graduate programs at both public and private institutions can adapt CURE-BxEco to mentor students through authentic scientific engagements using live video webcams from anywhere in the world. Furthermore, components of CURE-BxEco (or other CUREs) could also be extended outside of coursework to professional development modules that can aid in supporting a diverse workforce of practitioners in behavior, ecology, and evolutionary biology (e.g., research group members, animal caregivers). The CURE-BxEco curriculum has unparalleled flexibility in that it can be administered entirely online, without costly materials, and in variable timeframes (within 1 day, several days, or several weeks). As such, we also hope that CURE-BxEco will serve as a feasible means for research groups to incorporate community and workforce outreach into their teaching and service.

In looking ahead at the future of inclusive pedagogy across higher education, continued investigations are necessary to fully understand the mechanisms that make this type of curriculum effective, especially for historically excluded and minoritized students. In a universal sense, it strikes us as necessary to promote the routine collection of information on the intersectionality of students’ identities and experiences across higher education. Studies of student engagement, learning, and success should strive to collect information that encompasses the entire extent of students’ identities, not just sex assigned at birth and race but also dimensions of gender identity, sexuality, familial responsibilities, citizenship, visa status, religion, access to disposable income, and the necessity of secondary employment.

In addition, we see an urgent need to conduct international, multi-institutional, and cross-disciplinary assessments of the specific features of the university curriculum, mentorship culture, and overall academic environment that affect students’ senses of belonging to elucidate how and when students of historically excluded backgrounds can most benefit from opportunities to increase their science self-efficacy. To better understand the mechanisms that promote the effectiveness of CUREs, specifically, we would also promote longitudinal investigations into how experiences in CUREs across different educational settings (e.g., introductory first-year and advanced third- or fourth-year courses) affect the recruitment and retention of minoritized students, the ability to engage a larger number of students in research with CUREs compared with other mentorship models that may be less efficient (i.e., in terms of cost, resources, and time), and the professional impacts of CUREs relative to conventional field experiences. All such investigations are likely to be interdisciplinary as the students of ecology, evolution, and behavioral biology typically move across departmental divides (e.g., animal behavior, biological anthropology, human biology, evolutionary medicine). Finally, in practice, we must exert a concerted effort to harness the potential of CUREs to achieve a just, diverse, equitable, and thriving scientific community around the world.

## Supplementary Material

biae060_Supplemental_File

## Author Biography

Jake A. Funkhouser (jakefunkhouser@wustl.edu, jake.funkhouser@uzh.ch) is affiliated with the Department of Anthropology at Washington University, in St. Louis, Missouri, in the United States, and with the Department of Evolutionary Anthropology at the University of Zurich, in Zurich, Switzerland. Megan Gregory (meggregory@wustl.edu) is affiliated with the College Writing Program and with the Center for Teaching and Learning at Washington University, in St. Louis, Missouri, United States. Crickette Sanz (csanz@wustl.edu) is affiliated with the Department of Anthropology at Washington University, in St. Louis, Missouri, United States, and the Wildlife Conservation Society's Congo Program, in Brazzaville, Republic of Congo.
