# Supplementary material for: Promoting inclusivity in ecology, evolution, and behavioral biology education through course-based undergraduate research experiences
Source: Bioscience. 2024 Jul 30;74(8):567–76. doi: 10.1093/biosci/biae060 (PMC11367669; doi:10.1093/biosci/biae060)
Supplement: biae060_Supplemental_File [file biae060_supplemental_file.pdf]

BioScience

Supplementary Materials: *Promoting inclusivity in ecology, evolution, and behavioral biology education through course-based undergraduate research experiences*

Jake A. Funkhouser[1,2]*, Megan Gregory[3,4], & Crickette Sanz[5,6]

[1] Instructor and Research Associate, Department of Anthropology, Washington University in St. Louis, One Brookings Drive, Campus Box 1114, Saint Louis, MO USA 63130

[2] Postdoctoral Scholar, Department of Evolutionary Anthropology, University of Zurich, Winterthurerstrasse 190, Zurich, Switzerland 8057

[3] Associate Director for Faculty Programs and Services, Center for Teaching and Learning, Washington University in St. Louis, One Brookings Drive, Campus Box 1022, MO USA 63130

[4] Assistant Director and Senior Lecturer, College Writing Program, Washington University in St. Louis, One Brookings Drive, Campus Box 1096, MO USA 63130

[5] James W. and Jean L. Davis Professor in Arts and Sciences, Department of Anthropology, Washington University in St. Louis, One Brookings Drive, Campus Box 1114, Saint Louis, MO USA 63130

[6] Affiliate Scientist, Congo Program, Wildlife Conservation Society, B.P. 14537, Brazzaville, Republic of Congo

**Correspondence:*

Jake A. Funkhouser

jakefunkhouser@wustl.edu
Washington University in St. Louis
Department of Anthropology
One Brookings Drive
Campus Box 1114
Saint Louis, MO USA 63130

jake.funkhouser@uzh.ch
University of Zurich
Department of Evolutionary Anthropology
Winterthurerstrasse 190
Zurich, Switzerland 8057

Supplementary Materials

The Evaluation of the Effectiveness of CURE-BxEco

Across two semesters, we collated students' responses to beginning-of-semester reflections during the first week of class and end-of-semester reflections during the last week of class in *Behavioral Research at the Saint Louis Zoo (CURE-BxEco)*. The beginning- and end-of-semester reflections included items relating to personal perceptions about science where students were asked to rate on a Likert scale from 1 (strongly disagree) to 5 (strongly agree) if they believe science is a process, a body of knowledge, critical, creative, based on scientists' personal beliefs, systematic, replicable, produces knowledge that is certain and leads to ongoing research. Beginning- and end-of-semester reflections also included three scales that were previously validated by McBride et al. (2020): the Science Self-Efficacy (confidence scored increasingly from 1 to 10), Science Anxiety (agreement scored increasingly from 1 to 5), and Inclusion of Self in Science Scales (integration scored increasingly from 1 to 7). In addition to scale-rated items, both reflections asked students to respond to open-ended prompts about their previous experiences with the scientific process, sources of anxiety or confidence in scientific tasks, and aspects of the course they found most enjoyable or challenging. The exact language of our open-ended reflection questions and scale-rated items (McBride et al. 2020) are reported in <u>*Supplementary Tables S4-S5*</u>.

The data used in this investigation were compiled from reflections that students completed as a normal component of the curriculum. No identifying information was included as part of the data from these reflections. Further, to ensure confidentiality we did not collect demographic information. However, generally, it can be said that: one or more of the participants of this study were first-generation college students, identified as an underrepresented race and/or ethnic minority in the life sciences, identified as a member of the LGTBQIA+ community, and would likely not have otherwise had the opportunity to engage in independently designed research experiences. The research protocols described herein comply with the exempt categories of research in the Federal Policy for the Protection of Human Subjects (Common Rule 34 CFR 97.101b (1): data compiled on regular instructional activities to compare curricula).

To evaluate if the students' responses changed across the semester, we conducted non-parametric paired-sample Wilcoxon signed rank sum tests on the beginning- and end-of-semester composite scores. For each scale, composite scores were compiled by calculating the median of each student's responses to all the items of the same scale. Following the validated measures of McBride and colleagues (2020), we employed reverse coding when necessary; for example, responses for the science anxiety item "science is easy for me" were reverse-coded to match the direction of students' responses to "science makes me feel uncomfortable or nervous" (see McBride et al. 2020 for the complete details about the validation and reverse coding of these measures).

We collected a total of 31 complete beginning- and end-of-semester responses from students (out of 32 students enrolled). At the beginning of the semester, 100% of students

indicated that they planned to incorporate science and scientific approaches into their careers. Most of these students indicated that they planned to pursue medical or veterinary postsecondary degrees. However, only 50% of students indicated that any members of their families held careers in science and less than half (48%) reported that they had prior laboratory or research experience.

Students self-reported *science self-efficacy* scores significantly increased across the semester (median change = 2.25, z = 435, p < 0.01, CI = 1.75-2.75; *Supplementary Figure S2a*). Additionally, students' *perceptions about science* significantly changed towards stronger beliefs that science is an ongoing, uncertain, critical, creative, objective, and systematic process that produces a replicable body of knowledge (change in median scores = 0.50, z = 189, p < 0.01, CI = 0.01-0.75; *Supplementary Figure S2b*). However, our data do not indicate a significant change in students' self-reported *inclusion of self in science* (change in median scores = 0.01, z = 110.50, p = 0.53, CI = -0.50-1.00).

Students enumerated many sources of science-related anxiety in their open-ended responses at the beginning of the semester. Similarly, at the end of the semester, students reported many course experiences that improved their science self-efficacy and decreased their feelings of anxiety toward science (*Supplementary Table S2*). Overall, designing an independent project was the most cited source of anxiety *and* one of the most reported experiences that improved students' science self-efficacy. From the thematic coding of their short-answer reflections, many students also detailed how the structure of the course was impactful in improving their science self-efficacy and cultivating a sense of scientific community (*Supplementary Table S3*).

Overall, the students reported that the scaffolded independent research project and final manuscript, the collaborative nature of class activities, and working as a class to overcome the challenges of any individual project were impactful sources of science-self efficacy and helped to construct an inclusive community of scholars. Therefore, our limited assessment of the effectiveness of this curriculum indicates it was effective in (1) increasing students' science self-efficacy, (2) positively influencing students' beliefs about sciences, (3) fostering an inclusive science community, and (4) providing students with a sense of belonging in class and science more generally.

Supplementary Tables and Figures

Supplementary Table S1. Examples of student scholarship produced in CURE-BxEco

**Published in peer-reviewed journals**
Behavioral changes following alterations in the composition of a captive bachelor group of western lowland gorillas (*Gorilla gorilla gorilla*) (Gartland et al. 2018)
Black-tailed prairie dog affinity for sunlight and its potential animal welfare management implications (Eltorai and Sussman 2010)
The “visitor effect” and captive black-tailed prairie dog behavior (Eltorai and Sussman 2011)
**Presented in undergraduate research symposium**
Orangutan and siamang social relationships at the San Diego Zoo
Comparative patterns of elephant social behavior at Nashville and San Diego Zoos
The social behavior of a pregnant chimpanzee at the Saint Louis Zoo
The activity budget of an infant chimpanzee in response to visitors at the Saint Louis Zoo
**Projects conducted with remote data collection via live animal webcams**
Comparative social behavior of gentoo and king penguins at the Kansas City Zoo
The social behavior of captive dolphins at Clearwater Aquarium
The social behavior of domesticated donkeys living in a large herd at the Donkey Sanctuary
Mirroring and group homogeneity in Caribbean flamingos at the Saint Louis, Paignton, Memphis, Reid Park, and Maryland Zoos
**Projects conducted with remote data collection via video archives of animal behavior**
Alloparental care and infant access in chimpanzees at the Saint Louis Zoo
The function of peering in adult chimpanzees at the Saint Louis Zoo
The spatial variability of stereotypic behavior in three bear species at the Saint Louis and Kansas City Zoos
Orangutan wellbeing at the Saint Louis and San Diego Zoos

Note. Live animal webcam programs that have also been successful in other students' projects: Explore.org, Smithsonian's National Zoo and Conservation Biology Institute, Monterey Bay Aquarium, Edinburgh Zoo, and Taronga Zoo.

Supplementary Table S2. Sources of science anxiety as surveyed at the beginning of the semester and course experiences that improved science self-efficacy

Percent of Responses	Component of course
Sources of science anxiety identified at the beginning of the semester	
48 %	Independent project design (selection of research species, topic, or question)
31 %	Communicating results in journal-style manuscript
17 %	Conducting systematic behavioral observations and collecting data
14 %	Analyzing data and generating conclusions
14 %	Communicating results in conference-style presentation
10 %	Possibility of making (an) error(s)
7 %	Time and project management
Course experiences that improved science self-efficacy at the end of the semester	
30 %	Conducting systematic behavioral observations and collecting data
30 %	Independent project design (selection of research species, topic, or question)
23 %	Communicating results in journal-style manuscript
23 %	Analyzing data and generating conclusions
23 %	Consistent opportunities for feedback
23 %	Sense of scientific community and support
10 %	Communicating results in conference-style presentation
10 %	Working with peer in interobserver reliability activity

<u>Note</u>. *These responses were abbreviated from open-ended reflection questions. At the beginning of the semester, students were asked which aspects of the course they were "the most apprehensive, nervous, or anxious about." At the end of the semester, students were asked which aspects or experiences of the course had been the "most important influence on their confidence to perform or engage in science-related activities."*

Supplementary Table S3. Illustrative student reflections on the structure of the course, science self-efficacy, and community

Reflections on the structure of the course
**Applying the lecture material** to real-world problems challenged me the most, as I am so used to simply taking quizzes or exams.
I liked how we did **small assignments that led up to the final manuscript** throughout the course.
I think **looking, creating, and analyzing data** was something new for me because I do not take courses like this in college. This aspect was the most challenging, yet most rewarding to finish.
I was able to learn so much about the research process from start to finish and how one conducts research that I was never taught … I liked that there was both **freedom and structure** to the course: we had freedom in our topics, research plans, and what we wrote about yet there was also structure the whole way and support that was truly essential. I also really liked how **collaborative and supportive** the environment was.
Reflections on sources of science self-efficacy
I had very little confidence in the idea of writing a scientific manuscript by the end of the semester. I wasn't even entirely sure what a manuscript even was, nor did I know where to begin with my research. **Working on our manuscripts in bits and pieces** made the process seem less daunting. I also really enjoyed the constant feedback. **I now feel much more confident** in my ability to write a science-related manuscript.
The **independent nature of this course** helped me to improve my confidence about science. I had previously only had experience working on projects others had designed, so it was exciting and affirming to be so actively engaged in every stage of the process.
I enjoyed **creating something that was my own** and that I worked hard on. I liked that all of the effort that I invested in my project amounted to a polished manuscript of my work. I really felt like I contributed to something larger than myself and this course.
Reflections on senses of community
Seeing **everyone's projects evolve** was exciting!
I liked how **collaborative** and **supportive** the environment was.
I enjoyed listening to my **peers' ideas** and being **inspired by their creativity.**
Having such a **supportive class** made it easy to get help along the way and not shy away from asking for it, it also made me feel like all my progress was being acknowledged and commended.
I really enjoyed every part of this course and the research process because **I never felt like I was alone** and **I didn't have to figure out anything on my own**. It was fun to conduct my own research on a topic that I really loved and to explore my findings. I also loved hearing about everyone else's research and, really, just coming to class.
I appreciated hearing about what other students were studying and **how their research changed over the semester to overcome challenges**. While their topics may have been very different from mine, they were still super interesting, and the **sense of community** helped me tackle the course assignments with more enthusiasm.

Note. These responses were abbreviated from end-of-semester open-ended reflection questions where students were asked which aspects of the course they most enjoyed and which were most challenging. Bold emphasis was added to highlight pedagogical themes.

Supplementary Table S4. Beginning and end of semester course reflection scales

Perceptions of Science Scale

On a scale from 1 to 5 (where 1 = strongly disagree, 3 = no opinion, and 5 = strongly agree), please indicate how much you personally agree with the following statements:

Science is a process
Science is a body of knowledge
Knowledge produced during the scientific process is certain
Science is critical
Science is creative
Science is based in the researcher's personal beliefs
Science uses systematic, replicable methodology based on evidence
Science leads to ongoing research

Science Self-Efficacy Scale (McBride et al. 2020)

"On a scale from 1 to 10 (where 1 = not at all confident, 5 = moderately confident, and 10 = completely confident), please rate the level of confidence that you have in your ability to…

… apply scientific concepts and/or solutions to questions that are interesting to me
… read and understand peer-reviewed articles with quantitative data
… accurately evaluate the credibility of scientific information
… organize, analyze, and write about quantitative data and scientific information
… understand elements of research design and how they impact scientific conclusions
… develop a plan to investigate original research questions and test hypotheses
… justify inferences, predictions, and conclusions based on quantitative data
… solve problems using quantitative skills, including probability and statistics
… produce a scientific manuscript of original work
… give a presentation about original scientific work"

Science Anxiety Scale (McBride et al. 2020)

"On a scale from 1 to 5 (where 1 = strongly disagree, 3 = no opinion, and 5 = strongly agree), please indicate how much you personally agree with the following statements**:**

Science is easy for me
Science makes me feel uncomfortable or nervous"

Inclusion of Self in Science Scale (McBride et al. 2020)

"Looking at the graphic below, which image best describes your relationships with science?"

Series of "self" and "science" Venn diagrams (McBride et al. 2020).

Supplementary Table S5. Beginning and end of semester course reflection prompts

**Beginning-of-semester experience with science reflection prompts**
Before taking this class, how would you have defined "science"? Below, please provide a brief description of what you think "science" is.
Does anyone close to you work in science? What are your relationships with them, what do they do, and how do they work with science?
**Beginning-of-semester reflection prompts**
Please describe any experiences that you have had in other settings that have affected your confidence in your ability to perform or engage in science-related activities.
Of the experiences that you described above, please indicate which have been the most important influence on your confidence in your ability to perform or engage in science-related activities.
With the above definition of "science" in mind, do you think you will need or want to incorporate science, scientific approaches, or scientific conclusion into your career? If yes, how so?
Which aspects of this course are you most apprehensive, nervous, or anxious about?
**End-of-semester reflection prompts**
Have any aspects or experiences of this course impacted your confidence in your ability to perform or engage in science-related activities? If so, please list these aspects/experiences and describe how they impacted you.
Which of the aspects or experiences of this course have been the most important influence on your confidence in your ability to perform or engage in science-related activities?
Do you think you will need or want to incorporate science, scientific approaches, or scientific conclusions into your career? If yes, how so?
Which aspects of this course do you feel challenged you the most?
Which aspects of this course did you enjoy the most?

References for Supplementary Materials

Eltorai AEM, Sussman RW. 2010. The "visitor effect" and captive black-tailed prairie dog behavior. Der Zoologische Garten 79:109-120.

---. 2011. Black-tailed prairie dog affinity for sunlight and its potential animal welfare management implications. Animal Biology Journal 2:143-145.

Gartland K, McDonald M, Slade SB, White F, Sanz C. 2018. Behavioral changes following alterations in the composition of a captive bachelor group of western lowland gorillas (*Gorilla gorilla gorilla*). Zoo Biology 37:391-398.

McBride E, Oswald WW, Beck LA, Murray AV. 2020. "I'm just not that great at science": Science self-efficacy in arts and communication students. Journal of Research in Science Teaching 57:597-622.
